# Antiproliferative, Proapoptotic, Antioxidant and Antimicrobial Effects of *Sinapis nigra* L. and *Sinapis alba* L. Extracts

**DOI:** 10.3390/molecules23113004

**Published:** 2018-11-16

**Authors:** Valentina Boscaro, Luisa Boffa, Arianna Binello, Gabriella Amisano, Stefania Fornasero, Giancarlo Cravotto, Margherita Gallicchio

**Affiliations:** 1 Dipartimento di Scienza e Tecnologia del Farmaco, University of Turin, Via P. Giuria, 9, 10125 Turin, Italy; valentina.boscaro@unito.it (V.B.); luisa.boffa@unito.it (L.B.); arianna.binello@unito.it (A.B.); 2 Dipartimento di Scienze della Sanità Pubblica e Pediatriche, University of Turin, P.za Polonia 94, 10126 Turin, Italy; gabriella.amisano@unito.it (G.A.); stefania.fornasero@unito.it (S.F.)

**Keywords:** *Sinapis alba* L., *Sinapis nigra* L., ultrasound-assisted extraction, antiproliferative, proapoptotic, antimicrobial

## Abstract

High Brassicaceae consumption reduces the risk of developing several cancer types, probably due to high levels of glucosinolates. Extracts from *Sinapis nigra* L. (*S. nigra*) and *Sinapis alba* L. (*S. alba*) have been obtained from leaves and seeds under different conditions using ethanol/water mixtures because their glucosinolates are well accepted by the food industry. The EtOH/H_2_O 8:2 mixture gives better yields in glucosinolate amounts from ground seeds, mainly, sinalbin in *S. alba* and sinigrin in *S. nigra*. The highest antiproliferative activity in both non-tumor and tumor cell lines was induced by *S. alba* seeds extract. To evaluate whether the effect of *Sinapis* species (*spp*) was only due to glucosinolate content or whether it was influenced by the extracts’ complexity, cells were treated with extracts or glucosinolates, in the presence of myrosinase. Pure sinigrin did not modify cell proliferation, while pure sinalbin was less effective than the extract. The addition of myrosinase increased the antiproliferative effects of the *S. nigra* extract and sinigrin. Antiproliferative activity was correlated to Mitogen-Activated Protein Kinases modulation, which was cell and extract-dependent. Cell-cycle analysis evidenced a proapoptotic effect of *S. alba* on both tumor cell lines and of *S. nigra* only on HCT 116. Both extracts showed good antimicrobial activity in disc diffusion tests and on ready-to-eat fresh salad. These results underline the potential effects of *Sinapis spp* in chemoprevention and food preservation.

## 1. Introduction

The Brassicaceae family is composed of around 375 genera and about 3200 species [1], including broccoli, cabbage, cauliflower and mustard that originate from a common ancestral cabbage (*Brassica oleracea*), which still grows in Atlantic and Mediterranean areas [2]. Recent years have seen this family become a focus of growing interest as several epidemiological studies have demonstrated that high consumption levels of these vegetables reduce the risk of a great number of cancers, particularly cancers of the gastrointestinal tract, lung, bladder and prostate [3,4,5]. The ability of cruciferous vegetables to reduce cancer risk is not only due to the presence of fibre, carotenoids, lutein, flavonoids, phytosterols, folic acid and vitamin C, but also due to sulphur-containing secondary metabolites, namely glucosinolates, which are responsible for their bitter or spicy taste. Glucosinolates are composed of three moieties; a β-thioglucose moiety, a sulfonated oxime moiety and a variable side chain aglycone derived from an α-amino acid [6]. The biological activity of glucosinolates can be attributed to their hydrolytic products [2,4], which are derived from the action of myrosinase (β-thioglucosidase glucohydrolase, EC 3.2.3.1), an enzyme localized in districts where glucosinolates are not usually found. Glucosinolates and myrosinase come into contact when plant tissue is damaged, releasing unstable aglicones, which spontaneously rearrange into a variety of reactive compounds, mainly isothiocyanates (ITCs) [4,6]. This process can be mediated, in humans and animals, by the bacterial microflora of the gastrointestinal tract. However, the yield is lower in this process than when the process is mediated by plant enzymes [7]. The activity of myrosinase, whose stability differs by origin, can be preserved during the preparation of cruciferous vegetables, although it is often inactivated by a number of cooking methods, including boiling, steaming and microwaving at high power, thus, decreasing the bioavailability of the bioactive compounds. *Sinapis alba* (*S. alba*, white or yellow mustard) and *Sinapis nigra* (*S. nigra*, black mustard) are plants belonging to the Brassicaceae family that are extensively consumed by humans. They contain glucosinolate sinalbin (SNB) and sinigrin (SNG), respectively, which can be converted, by endogenous myrosinase, into benzyl isothiocyanate (BIT) first, and then, allyl isothiocyanate (AIT), which are responsible for the mustard flavor. It should be noted that black mustard myrosinase is more resistant to pressure and thermal treatment than the yellow mustard form. ITCs may exert their tumor-inhibiting properties via several, distinct mechanisms that involve the direct detoxification of carcinogens such as the prevention of the formation of carcinogen-induced DNA-adducts [8]. Sulforaphane shows both antioxidant and prooxidant activities, which can be utilized to eradicate or to prevent cancer [9]. Recently, it was demonstrated that phytochemicals can trigger autophagy in prostate [10], colon and breast cancer cells [11,12]. Sulforaphane also exerts an anti-angiogenic effect by inhibiting angiogenesis-activating transcription factors, such as HIF-1-alpha and c-Myc, and by reducing the production of matrix metallo-proteinase-2 and endothelial cell proliferation [13]. Several studies have highlighted the anti-inflammatory potential of sulforaphane, which is able to reduce inducible nitric oxide synthase and cyclooxygenase-2 expression as well as the secretion of tumor necrosis factor-alpha in cultivated macrophages [14].

AIT from SNG is also considered a food additive because of its flavoring and antimicrobial properties [15,16,17,18]. Recently, *Staphylococcus aureus (S. aureus)*, *Klebsiella pneumoniae (K. pneumonia)*, *Escherichia coli (E. coli)* and *Pseudomonas aeruginosa (P. aeruginosa)* strains were used to demonstrate antimicrobial activity of natural bioactive compounds [19]. The antibacterial activity of ITCs in humans was first described against *Helicobacter pylori (H. pylori)*, which is a known risk factor for gastric cancer; purified sulforaphane inhibited the growth and killed multiple strains of this bacterium in test tubes and tissue cultures, including antibiotic-resistant strains [20,21]. ITCs bactericidal effects on foodborne pathogens, including *Escherichia coli*, *Pseudomonas aeruginosa*, *Listeria monocytogenes* (L. *monocytogenes*) and *Staphylococcus aureus* have also been reported [3,22].

The aim of this study was to test the antiproliferative, proapoptotic, antioxidant and antimicrobial activities of a variety of extracts obtained from *S. alba* and *S. nigra* leaves and seeds, that had been cultivated in Piedmont, Italy. A variety of extraction conditions were used. In order to facilitate the selection of suitable samples on which to perform antiproliferative and antimicrobial tests, both glucosinolate amounts, through High Performance Liquid Chromatography (HPLC) analyses, and antioxidant activity, by testing 2,2-diphenyl-1-picrylhydrazyl radical (DPPH^•^) inhibition, were determined on all extracts. Potential chemopreventive activities were evaluated on both non-tumor [human telomerase reverse transcriptase-human mammary epithelial cell line (hTERT-HME1) and podocytes] and colon cancer cell lines (HCT 116 and HT-29). The antiproliferative activities of hydroalcoholic seed extracts were compared to those of the main glucosinolates, SNB (synonym sinapine glucosinalbate, SNP-GSB) in *S. alba* and SNG in *S. nigra*, to evaluate whether any activity present is only due to glucosinolate or whether it is influenced by the extracts’ complexity. We also investigated if the antiproliferative activity of these extracts is correlated to the modulation of the Mitogen-Activated Protein Kinase (MAPK) pathway, cell cycle progression and antioxidant activity. Finally, the antimicrobial activity of the hydroalcoholic seed extracts has been evaluated using both a disc diffusion test and a growth inhibition test on fresh salads, in order to combine their food preservation with chemoprevention potential.

## 2. Results

### 2.1. Extract Preparation

Fresh leaves and seeds of *S. alba* and *S. nigra* were extracted under different conditions (Table 1), in order to obtain the highest amounts of glucosinolates (SNG and SNB), using solvents that are suitable for the food industry.

It is well known that the mechanical effect of ultrasound (US) promotes the release of soluble compounds from plant bodies during extraction processes by disrupting cell walls, thus enhancing mass transfer and facilitating solvent access to cell content [23]. For extraction methods A and B, ground vegetable matrices were, therefore, immersed in a solvent (EtOH 96%; EtOH/H_2_O 8:2; H_2_O/β-cyclodextrin), and irradiated with US for 20 min (20 kHz, 150 W), before maceration at room temperature (r.t.), for 24 h. The HPLC analyses of *S. alba* and *S. nigra* leaf extracts show amounts of SNB sulfonate or glucosinalbate (GSB) and SNG that were lower than 1 mg/g (Table 1, method A), at w/w% extract yields of 2.54% and 1.86%, respectively. Extraction yields were 3.68% for white mustard and 3.43% for black mustard leaves. Ground seeds (different batches and different aliquots of the same batch), extracted according to method B, gave a mean yield value of 12.5% for *S. alba* and 11.1% for *S. nigra*., while method C, following the procedure described by Toribio et al. [24], granted 15.5% and 17.4% raw extract yields for white and black mustard seeds, respectively. Finally, an extraction protocol that involves the same conditions as method B was tested using 1.5% β-cyclodextrin (β-CD) in water, with the aim of achieving the selective complexation of glucosinolates [25].

Unfortunately, the obtained yields were lower than those derived from hot water extraction (10–12%). The EtOH/H_2_O 8:2 mixture gave the highest amount of GSB and SNG w/w% in the extracts (11.5 ± 1.4% and 17.7 ± 1.2%), with 14.4 ± 1.3 and 19.7 ± 1.8 mg per g being obtained from white and black mustard seeds, respectively. No SNG was found in the 1.5% β-CD-aqueous-solution black mustard seed extract, while a very low amount of GSB was obtained from white mustard. Despite literature data indicating the contrary, the aqueous extracts of mustard seeds were less rich in GSB and SNG w/w% than the EtOH/H_2_O 8:2 ones.

### 2.2. Antioxidant Activity

A DPPH^•^ assay was used to characterize the antioxidant capacity of all the extracts obtained, as it is one of the most accurate and responsive methods for analyzing vegetal extracts. The scavenging effect of the various extracts was screened to evaluate their DPPH^•^ inhibition percentage (I%), at a variety of concentrations. DPPH^•^ scavenging activity was expressed as the mean of the half maximal effective concentration (EC_50_) values ± standard deviation and was compared with the Trolox^®^ standard (data not shown). Trolox^®^ equivalents (TE), expressed as µmol/g of extract, were calculated based on EC_50_. Leaf and aqueous seed extracts showed the lowest antioxidant activity of all the samples screened, with EC_50_ values that were around a hundred times higher than Trolox^®^ (around 400 versus 3.94 ± 0.50 µg/mL), and a TE of nearly 40 µmol/g (Table 2). The EtOH/H_2_O 8:2 mixture furnished seed extracts with higher antioxidant capacity than the H_2_O extract. In particular, the highest antioxidant activity was found for the white mustard seed extract, which exhibited an EC_50_ of 56.6 ± 4.6 µg/mL and a TE of 278.1 ± 22.6 µmol/g.

### 2.3. Antiproliferative Activity

#### 2.3.1. Effect on the Cell Viability of Non-Tumor and Tumor Cell Lines

**Figure 1:**
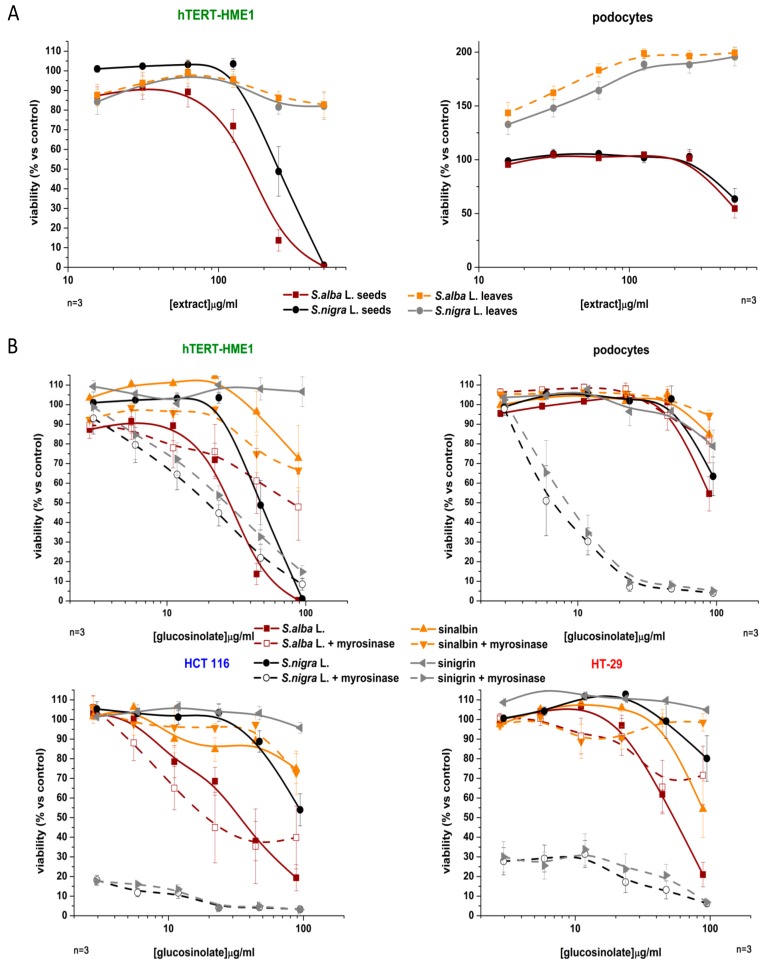
(**A**) Antiproliferative activity of a number of extracts of *S. alba* and *S. nigra* in hTERT-HME1 and podocytes. (**B**) Antiproliferative activity of sinalbin (SNB) and sinigrin (SNG), pure and obtained from the EtOH/H_2_O 8:2 seed extract of *S. alba* and *S. nigra*, in the presence and absence of myrosinase.

We compared the antiproliferative activity of extracts from the fresh leaves and seeds of *S. alba* and *S. nigra* (extracted in EtOH/H_2_O 8:2 mixture), on two non-tumor cell lines, hTERT-HME1 and podocytes (Figure 1A). Cells were treated with six different extract concentrations, in the 15.6–500 µM range, for six days, and then, cell viability was determined using the luminescent detection of cellular ATP content; each point was performed in triplicate.

The extracts obtained from the fresh leaves did not modify hTERT-HME1 viability, while those obtained from seeds were able to inhibit cell proliferation. The *S. alba* extract was more active than the *S. nigra* extract, and the IC_50_ values were 156.7 ± 1.08 µg/mL and 249.6 ± 2.12 µg/mL, respectively. Podocytes were sensitive to both seed extracts, though not to those obtained from leaves, which induced cell proliferation in a dose dependent manner. However, the seed extracts of both species were less effective on podocytes than on hTERT-HME1 and cell viability was reduced to 55% by *S. alba* and to 64% by *S. nigra*, at their maximum concentrations. Since seed extracts obtained with the EtOH/H_2_O 8:2 mixture showed the highest antioxidant activity and inhibited cell proliferation in non-tumor cell lines, their antiproliferative activity was also evaluated in two colorectal cancer cell lines, HCT 116 and HT-29. Furthermore, cell lines were treated with the principal glucosinolates present in these extracts, SNB in *S. alba* and SNG in *S. nigra.* The glucosinolates were used at the concentrations measured in each extract. Cells were treated with six different concentrations of glucosinolates, which were either pure or contained within the extracts, in the range of 2.77–88.5 µg/mL for SNG and 2.95–94.5 µg/mL for SNG, for six days.

Seed extracts from *S. alba* were more active than those from *S. nigra* in both cell lines. *S. alba* IC_50_ values were 33.69 ± 1.11 µg/mL in HCT 116 and 54.10 ± 1.06 µg/mL in HT-29 (corresponding to 189.83 µg/mL and 305.65 µg/mL of *S. alba* extract, respectively). HCT 116 cell viability was reduced to 55% and HT-29 viability to 81% at the maximum tested concentration of *S. nigra.* Extracts could not be tested at concentrations greater than 500 µg/mL, because those extracts were not soluble in the medium used for cell cultures (Figure 1B). Cell viability was not modified by pure SNG, while SNB inhibited cell proliferation despite being less active than the extract obtained from *S. alba* seeds. When cells were treated at the maximum extract concentration, HCT 116 and HT-29 viabilities were 55% and 33.5% lower, respectively, than when treated with pure SNB (Figure 1B).

SNG did not influence cell viability in hTERT-HME1, while SNB reduced it in a less significant manner than the *S. alba* extract did (Figure 1B). Podocytes are insensitive to both pure glucosinolates. This result may be partly explained by the fact that glucosinolates are not active as of themselves, but must be converted into ITCs by myrosinase. In order to evaluate whether SNB and SNG need enzymatic activation to exert their antiproliferative activity, one group of cells was treated with extracts and another with glucosinolates in the presence of myrosinase 0.1 U/mL (Figure 1B). The antiproliferative effects of SNG, whether pure or present in the *S. nigra* seed extract, increased in the presence of myrosinase. It inhibited hTERT-HME1 viability in a dose-dependent manner, with an IC_50_ value of 18.62 ± 1.20 µg/mL for SNG present in the extract (corresponding to 98.52 µg/mL of *S. nigra* extract, significantly lower than what was obtained without myrosinase, 249.6 µg/mL), and 26.00 ± 0.13 µg/mL for pure SNG. When myrosinase was added, podocyte viability was reduced by both pure SNG and the extract-borne analogue in a dose-dependent manner, with IC_50_ values of 8.73 ± 1.12 µg/mL and 7.17 ± 1.42 µg/mL (corresponding to 37.94 µg/mL of *S. nigra* extract), respectively. HCT 116 and HT-29 viability was reduced to 20% and 30%, respectively, at the lowest SNG concentrations in the presence of myrosinase. With regards to *S. alba*, the antiproliferative effect of the extract was greater than that of pure SNB. The addition of myrosinase to the medium did not modify the antiproliferative activity of SNB, whether pure or extracted from seeds.

#### 2.3.2. Effects on MAPK

**Figure 2:**
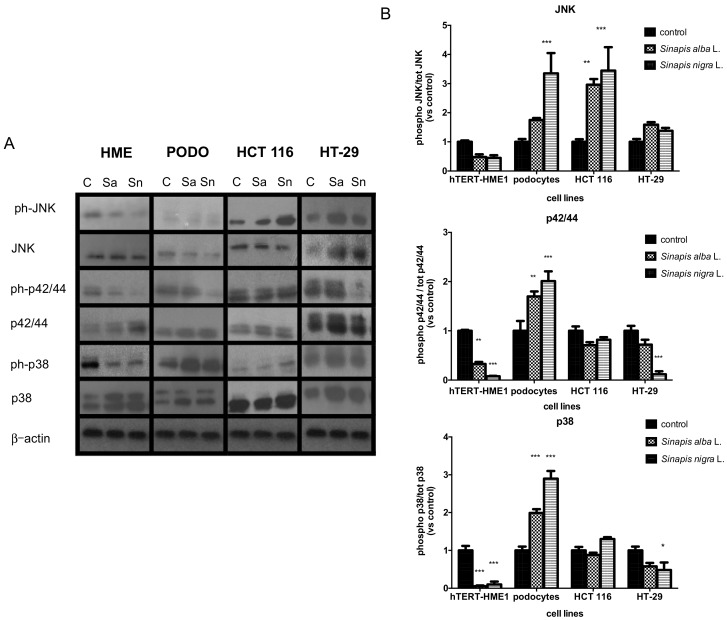
Effect of *S. alba* (Sa) and *S. nigra* (Sn) seed ethanolic extracts on the phosphorylation of p38, p42/44 and c-Jun N-terminal kinase (JNK) Mitogen-Activated Protein Kinases (MAPKs) at 30 min of treatment (**A**) Immunoblotting of one representative experiment of the two, at least, was performed. (**B**) Densitometric results, normalized to total protein, expressed in densitometric units with control = 1 [means ± standard error (SE) of at least two separate experiments].

In order to evaluate whether the antiproliferative activity of the seed extracts was correlated to the modulation of the MAPK pathway, cells were treated with the extracts at a variety of times (5–60 min), either at the IC_50_ values or at the maximum concentrations used in the proliferation assay if 50% proliferation inhibition was not reached (data not shown). Cells were processed with western blot analysis of the phosphorylated (ph−) and total forms of p38, p42/44 and c-Jun N-terminal kinase (JNK), as described in the methods section. The results reported are those obtained after treating cells for 30 min, as they were the most significant (Figure 2).

In hTERT-HME1, we observed a decrease in the phosphorylation of all the MAPKs analyzed, which was significant for p42/44 and p38, when cells were stimulated with *S. nigra* (*p* < 0.001) and *S. alba* (*p* < 0.01) (Figure 2B).

On the other hand, both the extracts had the effect of increasing MAPK phosphorylation in podocytes. *S. nigra* activated all the MAPKs analysed (*p* < 0.001), while *S. alba* was more active with p38 (*p* < 0.001) and p42/44 (*p* < 0.01) (Figure 2B). The only MAPK activated in HCT 116 by both extracts was JNK (*p* < 0.01 for *S. alba* and *p* < 0.001 for *S. nigra*). The results show that p42/44 was significantly dephosphorylated (*p* < 0.001) in HT-29 stimulated with *S. nigra*; p38 was dephosphorylated a lesser extent (*p* < 0.05).

#### 2.3.3. Effects on Cell Cycle

**Figure 3:**
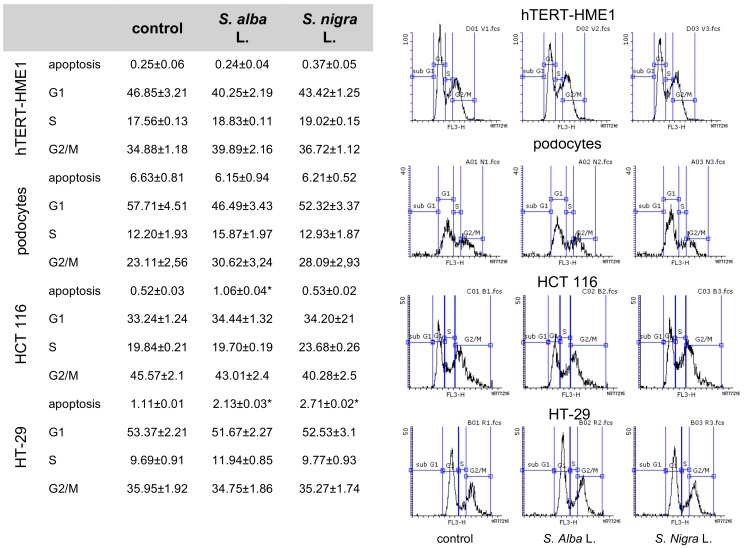
Effect of *Sinapis alba* and *Sinapis nigra* seed ethanolic extracts on cell cycle. Values are percentage of cells in each phase of the cell cycle, as a mean ± SEM (n = 3) (*p* < 0.05 compared with the control). G1: Gap 1; S: synthesis; G2: Gap 2; M: mitosis.

The inhibition of cell proliferation by *Sinapis spp* seed ethanolic extracts was only partly associated with cell cycle modulation (Figure 3). The treatment of hTERT-HME1 and podocytes, for 24 h with *S. alba* and *S. nigra* extracts, did not modulate the cell cycles. *S. alba* doubled the amount of cells in apoptosis in both tumor cell lines (*p* < 0.05 versus the control), while *S. nigra* was only effective in inducing apoptosis in HT-29 (*p* < 0.05 versus the control).

### 2.4. Antimicrobial Activity

**Figure 4:**
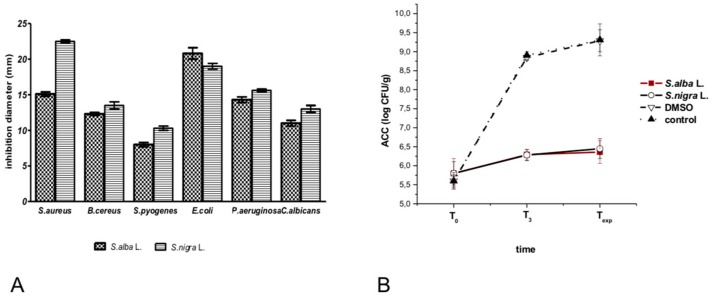
Antimicrobial activity of *S. alba* and *S. nigra* seed ethanolic extracts: (**A**) disc diffusion tests on several American Type Culture Collection (ATCC) strains (inhibition zones is expressed in mm); (**B**) bacterial growth trends in fresh-to-eat salad. Aerobic Colony Count (ACC) was measured at different times; T_0_: packaging date; T_3_: day 3; T_exp_: day 6.

Our results led us to choose *S. nigra* and *S. alba* seed EtOH/H_2_O 8:2 extracts for antimicrobial activity verification. The results of antibacterial tests on *E. coli*, *S. aureus*, *Bacillus cereus (B. cereus)*, *P. aeruginosa*, *Streptococcus pyogenes* (*S. pyogenes)* and *Candida albicans* (*C. albicans)* are shown in Figure 4A. Both black and white mustard seed ethanolic extracts were effective against all tested strains, showing inhibition zones of between 8 and 22.7 mm (20 mg/mL in DMSO), in disc diffusion tests, confirming the antimicrobial properties of these spices and their potential benefits in food preservation. DMSO was used as the solvent and did not show any activity. The greatest inhibition was exerted towards *S. aureus* and *E. coli* by *S. nigra* and *S. alba* (around 22 and 20 mm inhibition zones, respectively, 20 mg/mL conc.). *S. nigra* seed extracts showed stronger antimicrobial activity than *S. alba*, except against *E. coli*.

The antimicrobial activity of *S. nigra* and *S. alba* seed EtOH/H_2_O 8:2 extracts was also evaluated on ready-to-eat salad, in order to explore the possibility of extending shelf life. Untreated salad and salad treated with DMSO alone were examined as controls. Figure 4B shows bacterial growth trends of the treated and untreated samples which had been analyzed on the packaging date (T_0_), after three days (T_3_) and on the expiry date (T_exp_, day 6) [26]. At T_0_, all samples showed similar aerobic colony count (ACC), ranging from 5.6 to 5.8 log CFU/g with an average of 5.7 ± 0.02 log CFU/g. On the expiry date, salads treated with black and white mustard seed EtOH/H_2_O 8:2 extracts had ACC values of 6.36 ± 0.30 and 6.45 ± 0.26 log CFU/g, respectively, while the DMSO-sprayed and untreated samples showed ACCs of 9.29 ± 0.29 and 9.31 ± 0.42 log CFU/g, respectively. Analyses revealed 2.95 and 2.86 log decreases in bacterial growth in the salad packages treated with *S. nigra* and *S. alba* seed EtOH/H_2_O 8:2 extracts, respectively, as compared to control and DMSO treated samples. The distribution curves of 45 samples, analyzed as controls (same production batches as the sprayed samples), were compared with samples treated with the extracts and DMSO (data not shown). Results show that the values of total bacterial load in 45 packages of ready-to-eat salad, used as controls and examined at expiry date, approximate a normal distribution with a mean of 9.31 log CFU/g and a standard deviation of 0.42.

## 3. Discussion

Several epidemiological and case-control studies have lent their weight to the idea that the intake of cruciferous vegetables can reduce the risk of developing various types of cancer. This property is thought to be related to the presence of ITCs [5,7,27,28]. There are many examples of SNB and SNG extraction from varying species of mustard seed in the literature. Importantly, a number of solvents have been used, including aqueous acetonitrile [29], aqueous methanol [30], pure water and aqueous buffers [31,32]. In a number of protocols, a myrosinase inactivation step is carried out at a high temperature (100 °C), before extraction [24,33]. A comparison of a variety of extraction temperatures and solvents for *S. nigra* seed extraction has given SNG recovery values of between 1.6 and 2.35% (16–23.5 mg/g seeds) [31], while *S. alba* gave SNB yields of between 0.3 and 2.5% (3–25 mg/g seeds) [24,33].

In the present work, different parts (leaves and seeds) of *S. alba* and *S. nigra* have been extracted, using several methods and solvents, in order to find the best conditions under which to give samples with the highest amounts of glucosinolates. The US-assisted extraction of ground seeds using an EtOH/H_2_O 8:2 mixture provided extracts with the highest GSB and SNG, content (11.5 and 17.7% respectively), while the highest antioxidant activity was detected in *S. alba* seed extracts (278 TE µmol/g). Recent studies have described the DPPH^•^ radical scavenging activity of white mustard seed water extracts as giving inhibition values of 5.6%, 30.9% and 36.4 at concentrations of 1, 2 and 4 mg/mL, respectively [34], while black mustard seeds have shown a TE value of 4.80 ± 0.1 µmol/g [35]. The antioxidant activity of these water extracts was much lower than those found in our extraction protocols (even for water extracts), while comparable results have been obtained for *S. nigra* seed essential oil (EC_50_ value of 155 µg/mL) [36]. Selective β-CD complexation did not increase the antioxidant properties of seed extracts. This trend is more closely related to the presence of polyphenols, such as SNP, than to glucosinolate content. In fact, the SNP content is higher in white mustard seeds. Moreover, GSB hydrolysis generates *p*-hydroxybenzyl isothiocyanate (HBIT), a phenolic structure. Results reported in the literature assert that glucosinolate content appears to play a minor role in the antioxidant properties generally exhibited by cruciferous vegetables, since purified glucosinolates have exhibited only weak antioxidant properties [37].

Our data confirmed the antiproliferative effects that *Sinapis spp* have on tumor and non-tumor cell lines and the possible chemopreventive activity that these products present. We have observed that the extracts obtained from the leaves of *Sinapis spp* do not influence the viability of hTERT-HME1, but that they increase that of podocytes. On the other hand, both *Sinapis* seed extracts inhibit the cell proliferation of both non-tumor and tumor cell lines. This is not surprising, since the greatest glucosinolate concentrations are found in seeds (by HPLC analyses). In fact, the amount of SNG in the seed extracts is about 17.7%, compared to 1.86% in the leaf extracts for *S. nigra.* The average values for GSB are about 11.5% and 2.54% in the seeds and leaves of *S. alba*, respectively. These results are confirmed by literature data, which state that the glucosinolate concentration in seeds is ten times higher than that found in the leaves [38]. Moreover, myrosinase content is also higher in seeds than in leaves [39]. We cannot exclude the possibility that extraction methods completely inactivate myrosinase, meaning that the glucosinolates present in these extracts cannot be converted into the ITCs that are responsible for cell proliferation inhibition. Extracts obtained from *S. alba* seeds inhibit cell proliferation in a more significant manner than those obtained from *S. nigra*. Since the amount of glucosinolates in these two extracts is similar (17.7% for *S. nigra* and 18.9% for *S. alba* for the extracts used herein), the difference in the antiproliferative effects may be correlated to the different structure and physicochemical properties of the ITCs that originate from glucosinolate hydrolysis, namely HBIT from SNB, and AIT, a small volatile compound, from SNG. With regards to *S. alba*, the antiproliferative effect of the extract was greater than that of pure SNB. The activity of myrosinase in the extract may be conserved, at least in part, so that SNB can be hydrolyzed to HBIT. Nevertheless, pure SNB exerted a weak antiproliferative effect, while pure SNG did not modify cell viability. When myrosinase was added to the medium, the inhibitory effects of the *S. nigra* extract and pure SNG were comparable, meaning that the antiproliferative activity of the black mustard extract can be mainly attributed to SNG hydrolysis and AIT formation.

The difference between the antiproliferative activities of the black and white mustard extracts and their glucosinolates may also be ascribed to the presence of sinapine (SNP). SNP is a small molecular alkaloid extracted from the seeds of plants belonging to the cruciferous species [40]. Several studies have shown that SNP has various pharmacological activities, including antioxidant [41] and anti-inflammatory effects [42]. For example, SNP has inhibited the proliferation of Caco-2 cells in a dose-dependent manner and increased the cytotoxic effect of doxorubicin suppressing P-glycoprotein expression [40]. In our work, both pure SNB and the SNB present in the extract were in the form of the salt of sinalbin glucosinolate (GSB) with SNP, while pure tested SNG was the potassium salt (SNP present in black mustard extract was not stoichiometric). The amount of SNP was greater in *S. alba* than it was in the *S. nigra* seed extract (6.96% versus 4.5%, Table 1(A and B)).

In order to understand the mechanism behind the antiproliferative activity of these extracts, their effects on the activation of the MAPK pathway were investigated. Several studies in the literature have reported that both BIT and AIT treatment have increased the phosphorylation of MAPKs in a dose dependent manner and suppressed cancer cell growth [28,43]. We have observed that the modulation of MAPK activity is cell-dependent. In hTERT-HME1 and in HT-29, the antiproliferative activity of the extracts can be correlated to the considerable inhibition of p42/44 and p38 phosphorylation, even though a significant decrease in phosphorylation is only observed in HT-29 when cells are treated with *S. nigra*. Both *Sinapis* extracts activated all the MAPKs evaluated in podocytes, with *S. nigra* inducing a greater increase in phosphorylation. In HCT 116, the antiproliferative effects of *Sinapis* extracts can be explained by the activation of JNK, which directly regulates mitochondria-dependent apoptosis in a proapoptotic direction [44]. These results can be explained by MAPKs’ different modes of involvement in regulating proliferation and apoptosis in a cell specific manner. For example, even though p42/44 plays a crucial role as an antiapoptotic factor [44], a few recent studies have suggested that the activation of p42/44 is correlated to apoptosis, such as in human pancreatic cancer cells [43] and in ovarian cancer cells [13]. The p38 signaling pathway is also involved in a variety of cellular responses; it has been demonstrated that p38 promotes the death of melanoma cells [45], cardiomyocytes and fibroblasts [46]; additionally, this signaling pathway enhances survival and cell growth, such as in human cervical cancer cells [47] and in osteoblasts. Our cell cycle analysis showed that *Sinapis spp* was only able to induce apoptosis in the two tumor cell lines. There is a cell dependent effect, as these two cell lines also have different reactions to *Sinapis spp*: *S. alba* was able to drive both cell lines to apoptosis, while *S. nigra* was only proapototic for HT29.

Additionally, we tested the antimicrobial activity of both *Sinapis spp* seed extracts on several bacterial strains and on ready-to-eat salad. Of the bacterial strains tested, we observed that *S. nigra* and *S. alba* exerted the greatest inhibition towards *S. aureus* and *E. coli*, while the *S. nigra* seed extract showed stronger antimicrobial activity than *S. alba*, except against *E. coli*. However, data reported in 2014 by Aliakbarlu et al. on *S. alba*, gave quite different antimicrobial activity [34]; white mustard seed water extracts inhibited *Listeria monocytogenes* (15.3 mm inhibition zone at 150 mg/mL), while it was not active against *E. coli* and *B. cereus*. On the other hand, an *S. nigra* water extract showed good inhibition against several bacterial strains (for example, *L. monocytogenes*, *Salmonella typhimurium*, *E. coli*). In particular, the minimal inhibitory concentration found for *E. coli* was 10 mg/mL [35]. Disc diffusion tests on black mustard essential oil have shown similar antimicrobial properties against *S. aureus* (~19 mm inhibition zone at 10 mg/mL), while they were lower against *E. coli* (12 versus 20 mm). *B. cereus* was found to be the most sensitive to this oil (20–23 mm) [36]. Several papers have recently described the antibacterial activity of yellow and oriental mustard powder and their pure glucosinolates (SNB and SNG) in foods [48,49,50,51]. Tests were carried out against inoculated *E.coli* and *L. monocytogenes* in a variety of meats using non-deodorized and deodorized seed flour, seed extracts and pure glucosinolates applied directly to the meat surface or incorporated in the packaging. Both active and inactivated myrosinase powder samples were found to be active against inoculated pathogens, proving the key role that myrosinase-like activity has to play. The best results were obtained when the extended shelf life of foods allowed the bacterial conversion of glucosinolates to corresponding ITCs to occur. Oriental mustard showed greater inhibition than yellow mustard due to the greater volatility and moisture stability of that AIT shows over para-hydroxybenzyl isothiocyanate. Our tests on ready-to-eat salad have revealed that *S. nigra* and *S. alba* seed EtOH/H_2_O 8:2 extracts reduced bacterial growth in salad packages, as compared to control and DMSO treated samples, paving the way for a possible extension in their shelf life.

## 4. Materials and Methods

### 4.1. General Experimental Procedures

Analytical-grade ethanol (Carlo-Erba Reagenti, Milan, Italy), was used for extractions. The SNG ((–)–sinigrin hydrate, ≥ 99.0%, Chemical Abstracts Service (CAS) 3952-98-5, MW 397.46 for anhydrous base), reference sample was purchased from Sigma-Aldrich (Milan, Italy), while the SNB (HPLC grade > 99.0%, sinapine glucosinalbate, CAS 20196-67-2, MW 734.79), reference sample was obtained from Appen Lab S.r.l. (Turin, Italy). β-CD was kindly provided by Wacher Chemie (Munich, Germany). HPLC-grade acetonitrile (Carlo-Erba Reagenti), Milli-Q water and ammonium acetate (Sigma-Aldrich), were used for the HPLC mobile phases. The *S. alba* and *S. nigra* leaf samples were cultivated and characterized by Agroselviter (Prof. S. Nicola, Dipartimento di Agronomia, Selvicoltura e Gestione del Territorio, University of Turin, Turin, Italy), while the seeds were obtained from Martin Bauer S.p.A. (Turin, Italy; *S. alba* Cod. 1-879 Lotto A110014992/002; 25/10/12; *S. nigra* Cod. 1-880 Lotto A110014992/003; 25/10/12).

### 4.2. Extraction Procedures

#### 4.2.1. Method A

Leaves were immersed in EtOH 96% and ground in an immersion blender. The suspension was sonicated using a titanium immersion horn at 20 kHz (20 min, 150 W), and then kept at room temperature for 24 h for maceration. The suspension was filtered on paper in a Buchner funnel and the filtrate was dried under vacuum. The optimized plant/solvent ratio was 1:7.

#### 4.2.2. Method B

Liquid-nitrogen frozen seeds were subjected to fast cryogenic blender grinding and kept at −20 °C until extraction. Varying aliquots of seeds were poured into an appropriate solvent (Table 1), sonicated (20 kHz immersion horn at 150 W for 20 min) and kept at room temperature for 24 h for maceration. The suspension was filtered on paper in a Buchner funnel and the filtrate was dried under vacuum. The optimized plant/solvent ratio was 1:10.

#### 4.2.3. Method C

Liquid-nitrogen frozen seeds were subjected to fast cryogenic blender grinding and kept at −20 °C until extraction. Seeds were poured into water and the suspension was heated to 100 °C for 10 min, in order to inactivate myrosinase, and then kept at 70 °C for 4 h under silent conditions, according to the procedure described by Toribio et al. [24]. The mucilage was precipitated and centrifuged via the addition of ethanol (H_2_O/EtOH 1:1 ratio), while the supernatant was dried under vacuum. The plant/solvent ratio was 1:10.

### 4.3. HPLC Analyses

HPLC analyses were performed on a Waters 1525 Binary HPLC pump equipped with a 2998 photodiode array detector (PDA) [29]. Standard solutions of 0.22, 0.44, 0.66, 0.88, 1.1 and 7.1 mg/mL concentrations were prepared in water for the SNG calibration curve and a 30 µL amount of each was injected for HPLC analysis. Standard solutions of 0.115, 0.345, 0.69 and 1.15 mg/mL concentrations were prepared in water for the SNB calibration curve and a 20 µL amount of each was injected for HPLC analysis, using a Synergi Hydro RP C18 column (4 µm, 250 mm × 4.6 mm; Phenomenex). 0.025 M ammonium acetate in Milli-Q water (A) and acetonitrile (B) were used as the mobile phases. The gradient program started at 100% A was kept constant for 6.5 min, was taken up to 50% B at 22 min, kept constant at 32 min and finally taken to 100% B at 42 min, followed by a 100% B step from 42–48 min. The PDA three-dimensional (3D) data were collected between 210 and 400 nm, while the wavelengths at 228 and 242 nm were used to monitor the chromatograms.

### 4.4. Determination of Antioxidant Activity Using the DPPH^•^ Radical Scavenging Method

The radical scavenging ability of the extracts was evaluated using the stable, free-radical DPPH^•^, according to the method described by Brand-Williams, Cuvelier & Berset [52]. A DPPH^•^ solution (0.1 mM), was prepared to give an absorbance at 517 nm, in the 0.45-0.55 range. 700 µL aliquots of the DPPH^•^ solution were placed in cuvettes for the UV-Visible colorimetric assay. The reaction started when 700 µL of the diluted sample solutions were added to the cuvette containing the (DPPH^•^) solution. Mixtures were shaken vigorously and kept in the dark for 20 min at room temperature (time required to reach equilibrium). At this point, the UV absorbance of each sample was measured at 517 nm, measured against a pure methanol blank, on a Cary 60 UV-Vis spectrophotometer (Agilent Technologies, Santa Clara, CA, USA). Results are expressed as DPPH inhibition percentage (I %), which was calculated using the following equation:

I % = (Abs_DPPH•_ − Abs_sample_/Abs_DPPH•_) × 100.

The bleaching rate of DPPH^•^ was monitored in the presence of a number of solutions of Trolox^®^ (antioxidant standard), as well as in that of black and white mustard extracts in order to calculate the EC_50_ (amount of compound/extract necessary to decrease the initial concentration of DPPH^•^ to 50% at equilibrium). Radical scavenging activity in the Trolox^®^ solutions was measured at 1, 2, 4, 5, 7, 10, 12 and 18 µg/mL concentrations. Various concentrations of mustard extracts, between 5 and 2000 µg/mL, were also tested. Data analysis, the calculation of EC_50_ and Probit Regressions were performed using an algorithm in Microsoft Visual Basic 6.0 (Microsoft Corporation, Redmond, WA, USA) [53]. All samples were prepared in triplicate and DPPH^•^ radical scavenging activity was expressed as µg compound/dry extract per ml solution ± standard deviation. Trolox^®^ equivalents (TE) µmol/g of extract were calculated according to the EC_50_ values.

### 4.5. Cell Culture

HCT-116, HT-29 and hTERT-HME1 cell lines were purchased from the American Type Culture Collection. Immortalized human podocytes were kindly provided by Prof. Gianluca Miglio (University of Turin, Dept. Scienza e Tecnologia del Farmaco). HCT-116, HT-29 and podocytes were cultured in DMEM medium (Sigma-Aldrich) supplemented with 10% fetal bovine serum (FBS; Sigma-Aldrich). hTERT-HME1 cells were grown in Dulbecco’s Modified Eagle Medium: Nutrient Mixture F-12 (DMEM-F12) (Aurogene, Rome, Italy), supplemented with 2% FBS, 20 ng/mL epidermal growth factor (EGF), 10 µg/mL insulin and 100 µg/mL hydrocortisone (Sigma-Aldrich). All cell culture media were supplemented with 100 units/mL penicillin, 0.1 mg/mL streptomycin, 0.25 µg/mL amphotericin B and 2 mM glutamine (Sigma-Aldrich).

Extracts obtained from the leaves (method A) and seeds (method B) of *S. alba* and *S. nigra*, containing SNB and SNG, respectively, were dissolved in medium. Cells were seeded in complete medium (100 µL), at an appropriate density (1.500–2.000 cells/well), in 96-well plastic culture plates in triplicate. The following day, after serial dilutions in medium, which either contained 0.1 U/mL myrosinase (Sigma-Aldrich), or contained none, 100 µL of each product, in serum-free medium, was added to the cells using a multichannel pipette, while medium-only containing wells were used as controls. Plates were incubated at 37 °C in 5% CO_2_ for six days, after which cell viability was assessed using ATP content and the CellTiter-Glo^®^ Luminescent Cell Viability Assay (Promega, Italia Srl, Milano, Italy). All luminescence measurements (indicated as relative light units), were recorded on a Victor X4 multimode plate reader (Perkin-Elmer, Waltham, MA, USA).

### 4.6. Western Blot Analysis

Cells were grown in 6 well plates for 24 h and then starved and treated with extracts for different time periods (5, 15, 30, 60 min). All extracts were either used at the IC_50_ calculated from the previously performed antiproliferative assays, or at maximum concentration if 50% proliferation inhibition had not been reached. All extracts were dissolved in medium. Cells were washed twice with ice-cold PBS and lysed with RIPA buffer supplemented with a protease inhibitor cocktail (Sigma-Aldrich). The protein concentrations of cell lysates were determined using the Pierce^®^ BCA protein assay (ThermoScientific, Waltham, MA, USA), according to the manufacturer’s instructions. Cell lysates were then resolved using sodium dodecyl sulphate–polyacrylamide gel electrophoresis and transferred to polyvinylidene difluoride membranes (Biorad, Hercules, CA, USA). The primary antibodies used for immunoblotting were: anti-p42/44 MAP Kinase, anti-phospho-p42/44 Map kinase (Thr202/Tyr204), anti-phospho-p38 (Tyr 1068), anti-p38, anti-phospho-SAPK/JNK (Thr183/Tyr185) and anti-SAPK/JNK, all from Cell Signaling Technology (Danvers, MA, USA). The primary antibodies were diluted at 1:1000 in PBS containing 0.1% Tween-20 (PBS-T), and incubated overnight at 4 °C, except for anti-phospho-p42/44, which was diluted at 1:2000. The secondary antibody, used for all the primary antibodies reported above, was horseradish peroxidase-conjugated goat anti-rabbit immunoglobulin G (IgG) (Cell Signaling Technology), which was diluted at 1:2000 in PBS-T and incubated for 1 h at room temperature. In order to confirm the homogeneity of the protein loaded, the membranes were stripped and incubated with monoclonal anti-β-actin antibody (1:5000) (Sigma-Aldrich) for 30 min at room temperature, and subsequently, with horseradish peroxidase-conjugated goat anti-mouse IgG (1:2000) (Cell Signaling Technology), for 1 h at room temperature. Protein bands were visualized using Western Lightning Plus-ECL (Perkin Elmer, Waltham, MA, USA) [54]. Protein bands were quantified on the films by densitometry, using the ImageJ software (http://imagej.nih.gov/ij/, 1997–2015).

### 4.7. Cell Cycle

Cell cycles were analyzed using flow cytometry. 1.5 × 10^6^ cells were grown on a 10 cm plate for 24 h and then incubated with *S. alba* and *S. nigra* extracts for 24 h, either at the IC_50_ calculated from the previously performed antiproliferative assay, or at the maximum concentration if 50% proliferation inhibition was not reached. 1 × 10^6^ cells were then washed twice with ice-PBS and treated overnight with ethanol 70%. All samples were incubated with 100 µg/mL RNase (Sigma-Aldrich) for 1 h at 37 °C, and then with 100 µg/mL propidium iodide (Sigma-Aldrich), supplemented with 5 mM EDTA (Sigma-Aldrich), on ice. All fluorescence levels were detected using the flow cytometer BD AccuriTM C6 (Bd Biosciences, Erembodegem, Belgium). After doublet exclusion, an extended analysis of DNA content and calculations of the percentage of cells in each phase of the cell cycle were performed using Flowing Software 2.5.1 (released 4.11.2013).

### 4.8. Antimicrobial Diffusion Test

Antimicrobial diffusion tests were carried out to determine the antimicrobial activity of *S. alba* and *S. nigra* seed extracts, which had been solubilized in dimethyl sulfoxide (DMSO) (20 mg/mL). DMSO was selected because it does not show antimicrobial activity against the selected microorganisms and easily dissolves all extract components. Six microorganisms were selected for the bioassays: gram-positive strains *Staphylococcus aureus* ATCC 6538, *Streptococcus pyogenes* ATCC 12344 and *Bacillus cereus* ATCC 11778; gram-negative species *Escherichia coli* ATCC 8739 and *Pseudomonas aeruginosa* ATCC 10145 and, finally, opportunistic fungus *Candida albicans* ATCC 10231. Each bacterial strain was inoculated in Tryptic Soy Broth (TSB; Biolife S.r.l., Milan, Italy), and incubated at 30 °C at a variety of different times (for *Bacillus cereus*), and at 37 °C (for other bacteria), to obtain a concentration, in the exponential growth phase, of 10^6^ CFU mL^−1^. Fungal inoculum was prepared in Sabouraud Broth (Biolife S.r.l., Milan, Italy) and incubated for the time required at 25 °C to obtain a final concentration of 10^5^ CFU mL^−1^. About 100 µL of tested microorganism solution, obtained as described above, was spread over the entire surface of Mueller-Hinton Agar plates (Biomerieux, Craponne, France), using a spatula. Sterile paper discs (7 mm) were soaked in 0.2 mL of extract solution and placed on the inoculated plates at a suitable distance. After Petri dish incubation, at the required temperature and time, the growth inhibition zone diameter was determined in mm via measurement with a calliper. A control test, using a paper disc soaked in DMSO, was also carried out. All tests were conducted in triplicate.

### 4.9. Antimicrobial Activity in Salad

The antimicrobial activity of extracts was tested to evaluate their influence on fresh-cut salad microorganism growth. A total of one hundred and eighty samples were analyzed. Precisely four packs (200 g), for each of the 15 different production batches of the same salad brand were collected on packaging day (T_0_). Tests were carried out in triplicate for each production batch. After opening and sampling at T_0_, three packages in the same production batch were sprayed; one with 2 mL of *S. alba* seed extract solubilized in DMSO (20 mg/mL), another with *S. nigra* seed extract solubilized in DMSO (20 mg/mL) and a third with DMSO alone. A fourth was left untreated and was used as a control test. Salads were sealed and kept refrigerated at 4°C for later analysis (after three days, T_3_ and on the expiry date, day six, T_exp_). About 20 g of ready-to-eat lettuce was transferred to 180 mL of Tryptic Soy Broth (TSB, Biolife S.r.l.) in sterile stomacher bags and pummeled in a stomacher at 230 rpm for 2 min. Homogenized samples were serially diluted from 10^−1^ to 10^−9^ for subsequent plating on Plate Count Agar (PCA, Biolife S.r.l.) (International Organization for Standardization ISO 4833). Plate count agar was incubated for three days at 30 °C. Microbial counts were recorded as colony forming units per g (CFU/g) of sample. Data are shown as mean and standard deviation.

### 4.10. Statistics

The IC_50_ for each compound was calculated using GraphPad Prism 4.0 software. Where indicated, the results are given as the mean ± SE. Statistical analyses were performed using the two-tailed t-test with Bonferroni’s multiple comparison correction and the Instat program (GraphPad). Differences in means were considered significant at a significance level of 0.05 (*, *p* < 0.05; **, *p* < 0.01; ***, *p* < 0.001).

## 5. Conclusions

The correlation between a high Brassicaceae consumption and a reduction in the risk of developing several cancer types is well known. To obtain the highest concentration of glucosinolates from *S. alba* and *S. nigra,* considered the main responsible of their biological activity, we tested several methods of extraction, evidencing that the US-assisted extraction of grinded seeds using an EtOH/H_2_O 8:2 mixture afforded extracts with the highest glucosinolate (GSB and SNG) content.

Both extracts were able to inhibit cell proliferation, however, with different potency, evidencing that their antiproliferative activity were strictly correlated to the chemical structure of ITCs, deriving from their hydrolysis, and counter anions (SNP).

Both black and white mustard seed hydroalcoholic extracts were effective against *E. coli*, *S. aureus*, *B. cereus*, *P. aeruginosa*, *S. pyogenes* and *C. albicans* in disc diffusion tests, though showed the greatest inhibition towards *S. aureus* and *E. coli*, respectively. Fresh-cut salads sprayed with these two mustard extracts revealed significantly lower bacterial growth, as compared to untreated salads, paving the way for a possible extension in their shelf life.

These results confirm the antiproliferative, proapoptotic, antioxidant and antimicrobial properties of these spices and their potential benefits in chemoprevention and food preservation. In particular, *Sinapis* glucosinalates can be considered a good model for new drug development or could be used in combination with anticancer therapy because of their low toxicity and reduced side effects. Moreover, we could take advantage of *Sinapis* glucosinalates’ antimicrobial proprieties to reduce bacterial growth in salad packages, with an economic impact due to an increase in vegetables shelf life.

## Figures and Tables

**Table 1:** *Sinapis alba* (*S. alba*) and *Sinapis nigra* (*S. nigra*) extraction conditions, extraction yields and sinalbin sulfonate (GSB), sinigrin (SNG) and sinapine (SNP) percentages in the obtained extracts (EXT) and in the vegetal matrices (SAM). The values highlighted in the table are referred to the high GSB and SNG content.

*S. alba* Sample	Extraction Solvent (Plant/Solvent)	Extraction Method	Yield (w/w%)	GSB/EXT ^1^ (w/w%)	GSB/SAM ^1^ (mg/g)	SNP/EXT ^1^ (w/w%)	SNP/SAM ^1^ (mg/g)
Fresh leaves	EtOH 96% (1:7)	A	3.68	2.54	0.93	-	-
Seeds	**EtOH/H_2_O 8:2** **(1:10)**	**B**	**12.5**	**11.5**	**14.4**	**6.96**	**8.75**
Seeds	H_2_O/β-CD 1.5% (1:10)	B	11.9	0.30	0.35	0.20	0.24
Seeds	H_2_O (ppt EtOH) (1:10)	C	15.5	2.77	4.43	3.41	5.29
***S. nigra* Sample**	**Extracted Solvent (Plant/Solvent)**	**Extraction Method**	**Yield (w/w%)**	**SNG/EXT ^2^ (w/w%)**	**SNG/SAM ^2^ (mg/g)**	**SNP/EXT ^2^ (w/w%)**	**SNP/SAM ^2^ (mg/g)**
Freshleaves	EtOH 96% (1:7)	A	3.43	1.86	0.64	-	-
Seeds	**EtOH/H_2_O 8:2** **(1:10)**	**B**	**11.1**	**17.7**	**19.7**	**4.5**	**5.1**
Seeds	H_2_O/β-CD 1.5% (1:10)	B	10.1	-	-	0.32	0.32
Seeds	H_2_O (ppt EtOH) (1:10)	C	17.4	2.15	3.75	1.42	2.47

^1^ The %w/w GSB/EXT and SNP/EXT correspond to the percentage of GSB and SNP in the obtained extract, while the %w/w GSB/SAM and SNP/SAM correspond to the percentage of GSB and SNP in the original sample (leaves or seeds). ^2^ The %w/w SNG/EXT and SNP/EXT correspond to the percentage of SNG and SNP in the obtained extract, while the %w/w SNG/SAM and SNP/SAM correspond to the percentage of SNG and SNP in the original sample (leaves or seeds).

**Table 2:** Antioxidant activity of the various extracts compared with the Trolox^®^ standard expressed as EC_50_ values (mean ± standard deviation) and as µmol Trolox ^®^ equivalents (TE) per g of extract (EXT).

Sample	Extraction Solvent	EC_50_ ± SD (μg/mL)	TE/EXT (μmol/g)
Trolox^®^	-	3.94 ± 0.50	-
*S. alba* L. leaves	EtOH 96%	437.6 ± 37.2	36.0 ± 3.1
*S. alba* L. seeds	EtOH/H_2_O 8:2	56.6 ± 4.6	278.1 ± 22.6
	H_2_O/β-CD 1.5%	146.4 ± 13.0	107.5 ± 9.5
	H_2_O (ppt EtOH)	343.3 ± 23.7	45.9 ± 3.2
*S. nigra* L. leaves	EtOH 96%	372.9 ± 29.7	42.2 ± 3.4
*S. nigra* L. seeds	EtOH/H_2_O 8:2	194.7 ± 21.8	80.9 ± 9.1
	H_2_O/β-CD 1.5%	230.9 ± 15.1	68.2 ± 4.5
	H_2_O (ppt EtOH)	447.2 ± 48.7	35.2 ± 3.8
